# Supplementary material for: Impact of Gestational Diabetes and Hypertension Disorders of Pregnancy on Neonatal Outcomes in Twin Pregnancies Based on Chorionicity
Source: J Clin Med. 2023 Jan 31;12(3):1096. doi: 10.3390/jcm12031096 (PMC9917532; doi:10.3390/jcm12031096)
Supplement: Supplementary file 1 [file jcm-12-01096-s001.zip › jcm-2124411-supplementary.pdf]

Table S1. Neonatal outcomes according to maternal HDP and GDM status

Variables	No-GDM and no-HDP (n=1846)	HDP and GDM (n=106)	GDM and no-HDP (n=694)	HDP and no-GDM (n=150)	*P*-value
Gestational age at delivery, weeks	35.91 ± 2.05	36.04 ± 1.56	35.85 ± 1.95	35.45 ± 2.13	0.277
Preterm birth < 34 wk, n (%)	266 (14.4)	6 (5.7)	112 (16.1)	32 (21.3)	0.004
Mean birthweight, g	2354.4 ± 520.4	2375.3 ± 477.8	2380.7 ± 485.2	2244.1 ± 518.0	0.029
LGA, n (%)	139 (7.5)	10 (9.4)	60 (8.7)	10 (6.7)	0.676
SGA, n (%)	175 (9.5)	12 (11.3)	51 (7.4)	19 (12.7)	0.124
Intertwin birthweight discordance, %	12.1 ± 11.3	12.3 ± 9.4	11.5 ± 10.6	16.2 ± 12.4	0.002
Discordant twins, n (%)	177 (19.2)	12 (22.6)	50 (14.4)	25 (33.3)	0.002

± represent SD.

GDM, gestational diabetes mellitus, HDP: hypertension disorders of pregnancy, LGA: large for gestational age, SGA: small for gestational age.

Table S2. Neonatal outcomes among HDP-twin pregnancies complicated with GDM or not

Variables	HDP and GDM (n=106)	HDP and no-GDM (n=150)	*P*-value
Gestational age at delivery, weeks	36.0 ± 1.6	35.5 ± 2.1	0.075
Preterm birth < 34 wk, n (%)	6 (5.7)	32 (21.3)	<0.001
Mean birthweight, g	2375.3 ± 477.8	2244.1 ± 518.0	0.004
LGA, n (%)	10 (9.4)	10 (6.7)	0.416
SGA, n (%)	12 (11.3)	19 (12.7)	0.745
Intertwin birthweight discordance, %	12.3 ± 9.4	16.2 ± 12.4	0.048
Discordant twins, n (%)	12 (22.6)	25 (33.33)	0.189

± represent SD.

GDM, gestational diabetes mellitus, HDP: hypertension disorders of pregnancy, LGA: large for gestational age, SGA: small for gestational age.

Table S3. Association between HDP-twin pregnancies with GDM or not and neonatal outcomes.

Variables	HDP and GDM aβ/aOR (95%CI) *	*P*-value	HDP and No-GDM aβ/aOR (95%CI) *	*P*-value
Gestational age at delivery	0.30 (-0.09, 0.68)	0.134	-0.45 (-0.79, -0.11)	0.009
Preterm birth < 34 wk,	1.14 (0.72, 1.81)	0.571	1.49 (1.04, 2.15)	0.030
Mean birthweight	3.86 (-94.77, 102.49)	0.939	-127.30 (-212.24, -42.36)	0.003
LGA	0.99 (0.48, 2.04)	0.970	0.77 (0.39, 1.51)	0.439
SGA	1.62 (0.81, 3.25)	0.173	1.57 (0.94, 2.64)	0.088
Intertwin birthweight discordance	1.02 (-1.05, 3.12)	0.435	3.14 (2.12, 5.04)	<0.001
Discordant twins	1.72 (1.02, 2.91)	0.041	2.26 (1.56, 3.28)	<0.001

GDM, gestational diabetes mellitus, HDP: hypertension disorders of pregnancy, LGA: large for gestational age, SGA: small for gestational age. *Adjustment for maternal age, prepregnancy BMI, nulliparity, mode of conception and chorionicity.

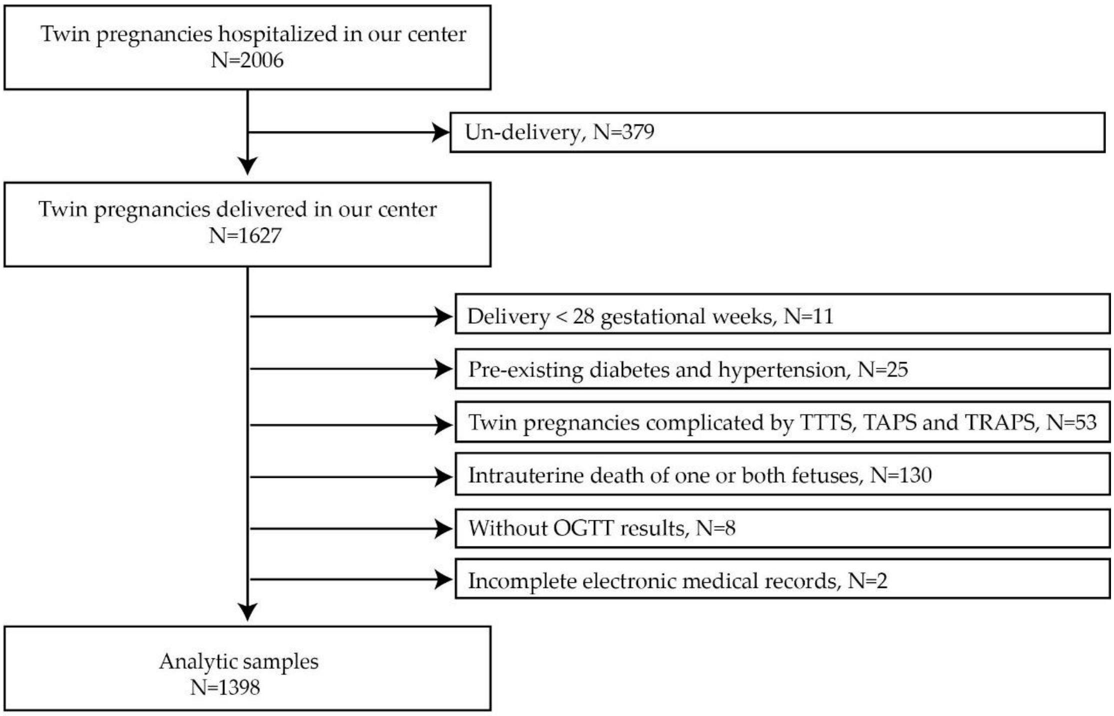

Figure S1. The flow diagram for participants selection process of this study
